# Alkali-Induced Phenolic Acid Oxidation Enhanced Gelation of Ginkgo Seed Protein

**DOI:** 10.3390/foods12071506

**Published:** 2023-04-03

**Authors:** Wei Zhang, Changqi Liu, Jing Zhao, Fengxian Guo, Jieyu You, Luyan Zhang, Yaosong Wang

**Affiliations:** 1 Department of Food Science and Engineering, Nanjing Forestry University, Nanjing 210037, China; 2 School of Exercise and Nutritional Sciences, San Diego State University, San Diego, CA 92182, USA; 3 Fujian Province Key Laboratory for Development of Bioactive Material from Marine Algae, College of Oceanology and Food Science, Quanzhou Normal University, Quanzhou 362000, China

**Keywords:** *Ginkgo biloba*, polyphenol, gelation, interaction

## Abstract

The effect of alkali-induced oxidation of three phenolic acids, namely gallic acid, epigallocatechin gallate, and tannic acid, on the structure and gelation of ginkgo seed protein isolate (GSPI) was investigated. A mixture of 12% (*w/v*) GSPI and different concentrations of alkali-treated phenolic acids (0, 0.06, 0.24, and 0.48% *w/w*) were heated at 90 °C, pH 6.0, for 30 min to form composite gels. The phenolic treatment decreased the hydrophobicity of the GSPI sol while enhancing their rheological properties. Despite a reduced protein solubility, water holding capacity, stiffness, and viscoelasticity of the gels were improved by the treatments. Among them, the modification effect of 0.24% (*w/v*) EGCG was the most prominent. Through the analysis of microstructure and composition, it was found to be due to the covalent addition, disulfide bond formation, etc., between the quinone derivatives of phenolic acids and the side chains of nucleophilic amino acids. Phenolic acid modification of GSPI may be a potential ingredient strategy in its processing.

## 1. Introduction

There is a constant increase in the demand for food nutrition as a result of the advancement of society and the increase in population. The consumption of plant protein is a sustainable alternative to animal protein in the event that animal protein is no longer sufficient to meet the needs of the global population [1]. The production of plant-derived protein is less environmentally harmful than the production of animal-derived protein [2]. Dated back to the Jurassic Period, *Ginkgo biloba* L. is one of the oldest tree species on the planet [3]. Ginkgo trees are widely planted in China and are highly valued for their leaves and seeds [4]. Ginkgo seeds are rich in nutrients and phytochemicals and are of high edible and medicinal value [5]. *Ginkgo biloba* L. has a significant amount of protein, containing between 10% and 20%, and is edible. Due to its amino acid composition and ratio, it is a relatively high-quality food protein with significant benefits for food development and utilization [6,7]. It is also important to consider the output of protein resources when evaluating their utilization. Ginkgo fruit is currently produced in China at a level of 60,000 tons per year [8], which accounts for 70% of the total production in the world [9], which brings a problem with their utilization. In addition to increasing plant-derived edible protein resources in order to decrease animal-derived protein dependence, extracting protein from plant sources is also in line with our goal of reaching peak carbon neutrality as well as contributing to sustainable agriculture and food production in compliance with the “carbon footprint” principle [10].

As stressed, Ginkgo seed proteins, which mainly comprise albumins and globulins, have a balanced amino acid profile and exhibit various biological functions, including anti-cancer and anti-inflammatory properties [11]. Aside from their antifungal properties, these proteins also possess free radical-scavenging properties [5]. Interestingly, the peptides derived from ginkgo seed proteins have the ability to inhibit α-glucosidase activity [12], as well exhibiting antiglycation and antioxidant properties, which contribute to the nutritional quality of foods [13]. To provide food with sensory properties and nutritional value, edible proteins are in the form of gels [14]. However, in comparison to other vegetable proteins, such as soybean and peanut proteins, ginkgo seed protein has low solubility and poor emulsifying and gelling properties, which greatly limit its application in the food industry [15,16].

There are many ways to modify proteins to enhance their gelling properties for the purpose of improving protein gelation [17]. One of them is polyphenol, which has become increasingly popular in recent years in the functional modification of food proteins [18]. Phenolics, with their diverse chemical properties and structures, are the most prevalent secondary metabolites found in plants. Within the food system, phenolic acids perform a number of important functions. Phenolic acids are the main coloring agents in plants, such as anthocyanins in strawberries and blueberries, and, therefore, phytophenolic acids are important natural pigments in the food industry [19]. Due to their special structure, phenolic acids possess good antioxidant properties, which makes them a popular natural antioxidant in food [20]. Moreover, studies have shown that phenolic acids engage in a variety of physiological activities [21]. Phenolic acids, a subgroup of phenolic compounds, are capable of interacting with proteins, leading to modifications in their physicochemical properties and structure [22]. These reactions involve several non-covalent interactions such as electrostatic interactions, hydrogen bonding, hydrophobic interactions, and van der Waals forces [23]. On the other hand, irreversible covalent binding can be achieved by oxidizing the phenolic compounds under the catalysis of polyphenol oxidase or alkali into quinones, which further react with the nucleophilic side chain groups of the amino acid residues [24]. To date, most studies on protein modification by polyphenols have focused on myofibrillar proteins. For example, various phenolic compounds have been shown to improve the gelation of porcine myofibrillar proteins [25]. Anqi Guo found that the formation of quinones by oxidizing gallic acid is conducive to the development of more elastic myofibrillar protein gels [26]. However, the influence of polyphenolic compounds on the gelation of plant proteins has not been thoroughly studied.

In this study, we selected three structurally distinct, water-soluble phenolic acids—gallic acid, epigallocatechin gallate, and tannin—to investigate the effect of alkali-induced phenolic oxidation on gelling properties of GSPI and elucidate the underlying mechanisms. Our findings demonstrate the application of phenolic species as natural protein modifiers for food product innovation.

## 2. Materials and Methods

### 2.1. Materials

Ginkgo seeds were obtained from a market in Xuzhou, Jiangsu, China. Gallic acid (GA, ≥99%), epigallocatechin gallate (EGCG, ≥95%), and tannic acid (TA, ≥98%) were supplied from Huazhong Haiwei Gene Technology Co., Ltd. (Beijing, China). All chemicals were purchased from Shanghai Lingfeng Chemical Reagents Co., Ltd. (Shanghai, China) or Sinopharm Chemical Reagent Co. Ltd. (Shanghai, China) and were of analytical grade.

### 2.2. Preparation of Ginkgo Seed Protein Isolate (GSPI)

Shelled and peeled fresh ginkgo seeds were dehydrated at 40 °C for 72 h, then ground and sifted through an 80-mesh. The resulting powder was subjected to defatting with n-hexane (1:8, *w/v*) thrice. The defatted flour was mixed with deionized water in a ratio of 1:9 (*w/v*), and the pH was adjusted to 9.0 using 1 N NaOH. The mixture was stirred for 2 h at room temperature (RT, 22 °C). The supernatant was collected after centrifugation at 5000× *g* for 15 min at 4 °C. The supernatant was collected and further adjusted to pH 4.4 using 1 N HCl, then centrifuged under the same conditions. The resulting precipitate was dissolved in deionized water and neutralized to pH 7.0 using 1 N NaOH. The resulting GSPI was freeze-dried and stored at 4 °C until further analysis.

### 2.3. Preparation of GSPI-Phenolic Acid Sols

A 24 % (*w/v*) GSPI sol was made by stirring the protein with deionized water for 4 h at RT. The GSPI dispersion was refrigerated at 4 °C overnight and brought to RT before use.

GA, EGCG, and TA were dissolved in deionized water to different concentrations (0.12%, 0.48%, 0.96% *w/v*), and their pH was adjusted to 8.0 using 1 N NaOH. Oxygen was bubbled through the solution with magnetic stirring for 4 h. After oxidation, the pH of the solution was adjusted to 7.0 using 1 N HCl.

The GSPI sol was mixed with the phenolic acid solutions of different concentrations at a 1:1 (*v/v*) ratio. The mixture was thoroughly stirred and immediately adjusted to pH 6.0. The final GSPI concentration was 12% (*w/v*), while the concentrations of phenolic acids were 0%, 0.5%, 2.0%, and 4.0% (*w/w*) of the protein content. The mixture was stirred for 4 h before analyses.

### 2.4. Solubility of GSPI-Phenolic Acid Sols

The biuret method was used to determine the soluble protein concentration. The GSPI-phenolic acid dispersions were adjusted to a protein content of 10 mg/mL using 50 mM phosphate buffered saline (pH 6.0) and centrifuged for 10 min (20 °C, 5000× *g*). The supernatant (1 mL) was mixed with four milliliters of biuret reagent and incubated in a water bath at 30 °C for 30 min. The absorbance was determined at 540 nm using phosphate-buffered saline as a reference [16].

The protein solubility was calculated using the method described by Cao and is expressed as follows [27]:

(1) $$\mathrm{Protein}\;\mathrm{solubility}\;\left(\%\right)=\frac{\mathrm{Protein}\;\mathrm{content}\;\mathrm{in}\;\mathrm{the}\;\mathrm{supernatant}\;}{\mathrm{Protein}\;\mathrm{content}\;\mathrm{in}\;\mathrm{the}\;\mathrm{original}\;\mathrm{dispersion}}\times 100\%$$

### 2.5. Surface Hydrophobicity of GSPI-Phenolic Acid Sols

The hydrophobicity of the protein surface was evaluated by employing 1-anilinonaphthalene-8-sulfonic acid (ANS) fluorescent probes [28]. GSPI-phenolic dispersion was diluted to 0.1, 0.2, 0.3, 0.4, and 0.5 mg/mL with 50 mM sodium phosphate buffer (pH 6.0), and 8 mM ANS was added. The fluorescence signal was measured using a microplate reader after 15 min of incubation in the dark. The excitation and emission wavelengths were 365 nm and 484 nm, respectively, and the slit width was 5 nm. The hydrophobicity of the protein surface was determined by calculating the slope of the linear curve obtained by plotting fluorescence intensity against protein concentration.

### 2.6. Particle Size and ζ-Potential of GSPI-Phenolic Acid Sols

GSPI-phenolic acid sols were diluted to a protein concentration of 2 mg/mL with 50 mM sodium phosphate buffer (pH 6.0). A Zetasizer Nano ZS90 (Malvern Instruments Co., Ltd., Malvern, UK) was used to determine the particle size and *ζ*-potential of the diluted samples [29].

### 2.7. Intrinsic Fluorescence

The intrinsic fluorescence was determined according to Zhang [16] with modifications. The GSPI-phenolic acid sols were diluted to 1 mg/mL using 50 mM phosphate buffer (pH 6.0). The emission spectrum was recorded between 320 and 400 nm using a microplate reader at an excitation wavelength of 295 nm with a scanning speed of 1 nm/s. The blank used for this measurement was 50 mM phosphate buffer at pH 6.0.

### 2.8. Sodium Dodecyl Sulfate-Polyacrylamide Gel Electrophoresis (SDS-PAGE)

SDS-PAGE was performed using a 12% separation gel and a 5% concentrating gel. Constant voltages of 50 mA and 90 mA were applied for the concentrating and separation gels, respectively. The gels were stained by Coomassie Brilliant Blue R250, followed by de-staining using 7.5% glacial acetic acid until the gel background was removed.

### 2.9. Dynamic Rheological Analysis

The rheology of GSPI-phenolic acid sols was determined with slight modifications based on Sriprablom et al. [30]. All rheological measurements were performed at RT using a Discovery Hybrid Rheometer 1 (TA Instruments, New Castle, DE, USA) equipped with a 40 mm diameter aluminum plate and a 1 mm gap between the Peltier plate and the fixture. A circulating cooling system was used to ensure precise temperature control of the sample.

Time sweep oscillatory tests were conducted to measure the storage modulus (G′) and loss modulus (G″) of each sol sample. Two milliliters of the sol was placed on the rheometer, and silicone oil was added to prevent water evaporation. A frequency of 1 Hz and a strain at 1% were used to maintain the strain within the viscoelastic region for all specimens [31]. The samples were subjected to a temperature sweep range of 25 °C to 90 °C at a rate of 5 °C/min and then held at 90 °C for 10 min before cooling to 25 °C at a rate of 5 °C/min.

### 2.10. Preparation of GSPI-Phenolic Acid Gels

Six milliliters of sealed GSPI sols were heated in a 90 °C water bath for 30 min. The samples were cooled to room temperature (RT) and kept at 4 °C overnight. Gels were equilibrated at RT for 2 h before subsequent analyses.

### 2.11. Water Holding Capacity (WHC) of GSPI-Phenolic Acid Gels

The determination of the WHC of the gels followed the method described by Wang [32]. Approximately 1.0 g of gel sample were subjected to centrifugation at 3000× *g* for 30 min at RT. The WHC was calculated as the percentage of the weight of the centrifuged gel to the weight of the original gel.

### 2.12. Texture Profile Analysis (TPA) of GSPI-Phenolic acid Gels

TPA test was conducted using a TA.XT Plus texture analyzer (Stable Micro Systems Ltd., Godalming, UK) with a P/0.5-cylinder probe [33]. The gels tested by TPA were cylindrical with a diameter and height of 20 mm each. Double compression tests were performed under the conditions of 2 mm/s speed, 7 mm distance, and 5 g trigger force.

### 2.13. Scanning Electron Microscopy (SEM)

The microstructure of the gels was determined using a scanning electron microscope (FEI Company, Eindhoven, The Netherlands). The samples were mounted on bronze stubs and coated with gold to ensure electrical conductivity. The specimens were examined at an acceleration voltage of 15 kV.

### 2.14. Fourier-Transform Infrared (FT-IR) Spectroscopy

Gel samples were lyophilized prior to FT-IR measurements. Potassium bromide was added to the sample powder, dried to constant weight, ground into a uniform powder, and pressed into a transparent sheet by a tablet press. An FT-IR spectrometer (VERTEX 80 V, Bruker, Germany) was used to scan the samples in the wavenumber range of 4000 to 400 cm^−1^, where the wavenumber precision was 0.01 cm^−1^ and the resolution was 4 cm^−1^.

### 2.15. Statistical Analysis

All experiments were repeated at least twice, and the results were expressed as the mean ± standard deviation. The data were analyzed using Statistix 9.0 (Analytical Software, Tallahassee, FL, USA). The differences between groups were analyzed by analysis of variance (ANOVA), followed by Fisher’s least significant difference (LSD) test at a significant level of α = 0.05.

## 3. Results and Discussion

### 3.1. Effects of Oxidized Phenolic Acids on Properties of the GSPI Sols

**Figure 1:**
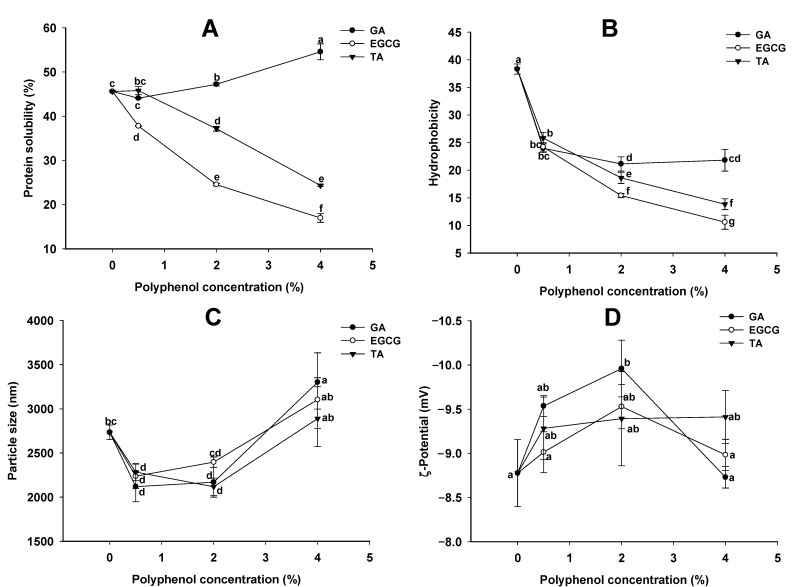
Protein solubility (**A**), surface hydrophobicity (**B**), particle size (**C**), and ζ-potential (**D**) of the phenolic acid-modified ginkgo seed protein at different concentrations (0, 0.5, 2.0, 4.0%, *w/w*) of gallic acid (GA), epigallocatechin gallate (EGCG), and tannic acid (TA). Values sharing no common letters differ significantly (*p* < 0.05).

Solubility is an essential function and a determinant of other functionalities of proteins [34]. As shown in Figure 1A, the solubility of GSPI decreased with increasing concentrations of EGCG and TA, while it increased with GA treatment. Polyphenol–protein interactions are known to alter protein structures and cause the formation of complex aggregates, which explains the decreased protein solubility caused by EGCG and TA treatments [35]. In addition, the covalent protein–quinone–protein cross-linking may also contribute to the decreased solubility of GSPI. The increased protein solubility by GA treatment was also reported for myofibrillar protein and β-lactoglobulin [35,36,37]. GA-induced partial secondary and tertiary structural rearrangements were likely responsible for the increased protein solubility [37].

The phenolic acid treatments significantly reduced the surface hydrophobicity of GSPI, with EGCG and TA being more potent than GA (Figure 1B). Aewsiri also reported that phenolic modification introduced hydrophilic -OH and -COOH groups and reduced the surface hydrophobicity of cuttlefish skin gelatin [38]. In addition, protein aggregation induced by phenolic acid may have prevented the ANS probe from accessing hydrophobic groups [39]. The lower hydrophobicity of EGCG-GSPI and TA-GSPI may be due to the higher affinity of EGCG and more abundant binding sites of TA [35].

As shown in Figure 1C, with an increase in the phenolic acid concentration, the average particle size of GSPI first decreased and then increased. The electrostatic repulsions between the protein molecules could not resist the hydrophobic interaction and van der Waals force at pH 6.0, leading to the aggregation of GSPI particles [33]. The decreased particle size at low concentrations of phenolic acids may be due to the reduced surface hydrophobicity (Figure 1B) and, thus, a mitigated hydrophobic interaction between the protein molecules. At higher phenolic concentrations, the cross-linking of GSPI by phenolic acids resulted in the formation of larger protein aggregates [40].

The phenolic acid treatments had little effect on the zeta potential of the GSPI (Figure 1D). The only significant change was observed when 2% GA was added, which reduced the negative surface charges of the protein. The decreased zeta potential could be the result of the charge shielding effect that occurred when GA interacted with GSPI [41].

### 3.2. Intrinsic Fluorescence of the GSPI Sols

**Figure 2:**
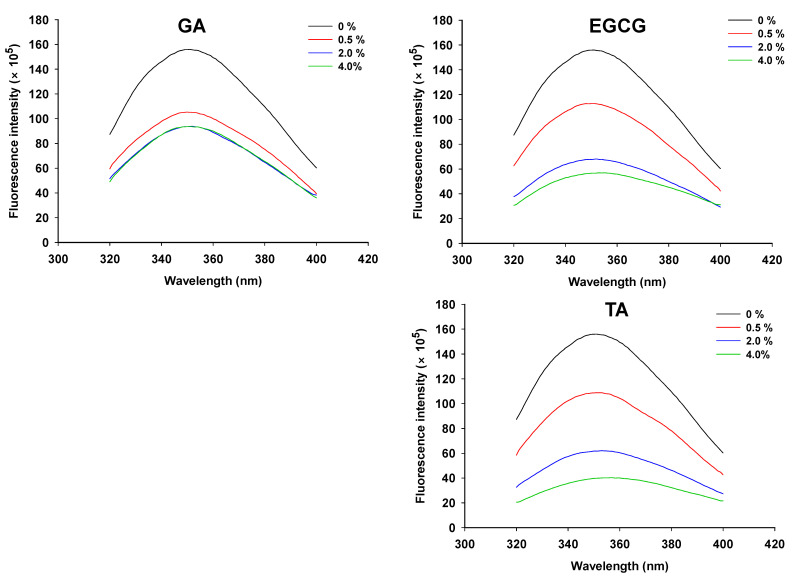
Intrinsic fluorescence spectra of the phenolic-modified ginkgo seed protein at different concentrations (0, 0.5, 2.0, 4.0%, *w/w*) of gallic acid (GA), epigallocatechin gallate (EGCG), and tannic acid (TA).

Features of intrinsic tryptophan fluorescence spectra are often used to study protein tertiary structure [26]. According to Figure 2, the fluorescence intensity of phenolic acid-treated GSPI was reduced compared to the control group. This suggests that phenolic acids disrupted the protein tertiary structure and exposed buried tryptophan residues to a more hydrophilic environment, thus quenching the fluorescence of GSPI [42]. Tryptophan fluorescence was suppressed in a dose-dependent manner by phenolics, indicating that GSPI gradually unfolded with increasing phenolic content. Furthermore, the covalent binding between GSPI and quinone derivatives of phenolic acids promoted changes in protein structure [43].

### 3.3. SDS-PAGE of Phenolic Acid-Treated GSPI

**Figure 3:**
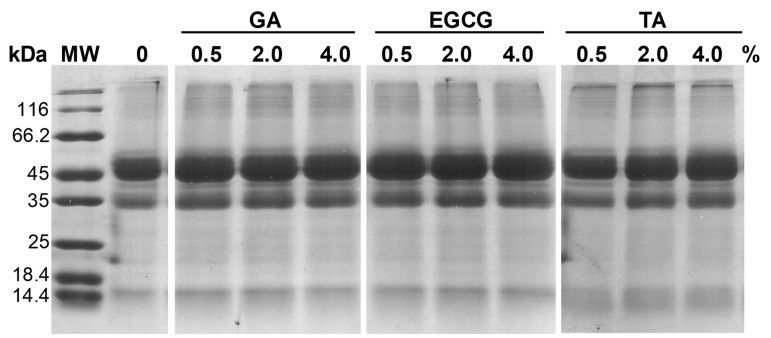
SDS–PAGE patterns of the phenolic acid-modified ginkgo seed protein at different concentrations (0, 0.5, 2.0, 4.0%, *w/w*) of gallic acid (GA), epigallocatechin gallate (EGCG), and tannic acid (TA). MW: molecular weight.

The protein profile of ginkgo seeds has not been fully characterized, with the exception of the 11S gennacin and some minor biologically active proteins [16]. SDS-PAGE analysis of untreated and phenolic acid-treated GSPI was performed, and the results are shown in Figure 3. The phenolic acid treatments resulted in little change in electrophoretic patterns of the GSPI, which was similar to the results of Anqi Guo in their study of phenolic compound-modified myofibrillar proteins [26]. This indicates that the molecular interactions between the phenolic acids and GSPI were mainly via non-covalent bonds, which were disrupted by SDS. However, the presence of large protein aggregates at the top of the gel, particularly in TA-treated GSPI, indicated protein cross-linking via covalent bonds. The larger molecular weight and more active sites of TA might have enabled more efficient protein cross-linking [35].

### 3.4. Rheological Characteristics of GSPI-Phenolic Acid Gels

**Figure 4:**
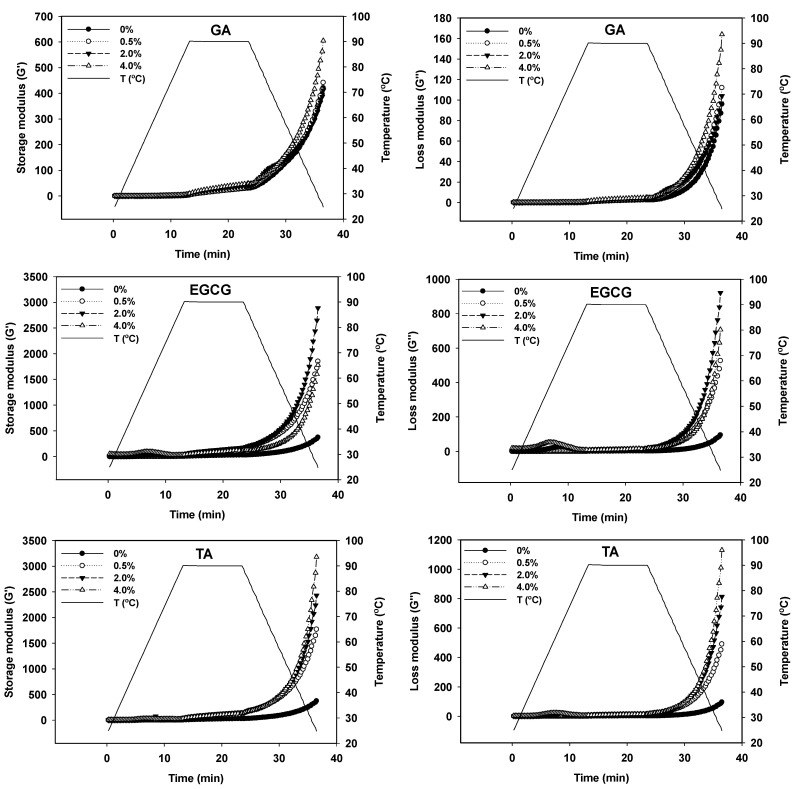
Storage modulus (G′) and loss modulus (G″) of the phenolic acid-modified ginkgo seed protein gels at different concentrations (0, 0.5, 2.0, 4.0%, *w/w*) of gallic acid (GA), epigallocatechin gallate (EGCG), and tannic acid (TA).

During the heating process, the GSPI undergoes denaturation, aggregation, cross-linking, and finally, gel formation. To assess the impact of phenolic compounds on the dynamic viscoelastic behavior of GSPI during thermal gelation, the storage modulus (G′) and loss modulus (G″) of the samples were measured. As shown in Figure 4, the G′ and G″ of the phenolic acid-treated GSPI barely changed when the samples were heated from 25 °C to 90 °C. When the samples were held at 90 °C for 10 min, the G′ and G″ slowly increased as a result of exposed hydrophobic and sulfhydryl groups. During the cooling stage, the viscoelastic properties of the gels increased rapidly due to strengthened hydrogen bonding. GA exhibited little effect on the rheological properties of the samples, while the 4% TA- and 2% EGCG-treatments increased the G′ by 748% and 670%, respectively. The G′ and G″ of the untreated GSPI gel eventually reached 380 and 100, respectively. Anqi Guo also reported that the triphenol EGCG was more effective in enhancing the elastic parameter of myofibrillar protein gels than the monophenol GA [26]. Quinone derivatives of polyphenols could facilitate GSPI gelation by promoting SH/SS exchange and by having nucleophilic addition reactions with the amino and sulfhydryl groups of the GSPI [24]. However, extensive modifications, such as the case of 4% EGCG, may result in the blocking of thiol groups and thus hinder protein–protein interactions [18].

### 3.5. Appearance and WHC of GSPI-Phenolic Acid Gels

**Figure 5:**
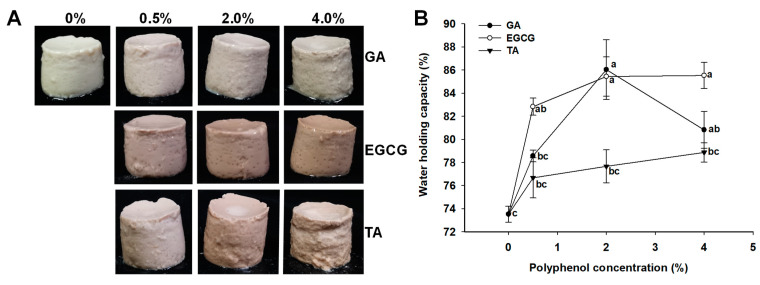
Appearance (**A**) and water holding capacity (**B**) of the phenolic acid-modified ginkgo seed protein gels at different concentrations (0, 0.5, 2.0, 4.0%, *w/w*) of gallic acid (GA), epigallocatechin gallate (EGCG), and tannic acid (TA). Values without a common letter were significantly different (*p* < 0.05).

The phenolic acid-treated GSPI gels were formed at three phenolic acid concentrations of 0.5%, 2.0%, and 4.0%. Appearances of the gels are shown in Figure 5A. The control gel without phenolic acid was milky white. The addition of oxidized phenolic acids, particularly EGCG, gave the GSPI gels a brown hue.

The WHC of a gel measures the quantity of water retained in the gel matrix after centrifugation. As shown in Figure 5B, TA had no significant effect on WHC, although a trend towards enhanced WHC was observed with an increasing concentration of TA. EGCG and GA, on the other hand, increased the WHC of GSPI at ≥0.5% and ≥2.0% concentrations, respectively. The enhanced WHC may be attributed to phenolic acid-induced protein cross-linking and the formation of a stronger gel network that can imbibe more water [44]. Nevertheless, high concentrations of polyphenols could lead to excessive protein denaturation and aggregation, which may hinder water entrapment [45], a trend observed when 4.0% EGCG was added.

### 3.6. Texture Profile Analysis of GSPI-Phenolic Acid Gels

**Figure 6:**
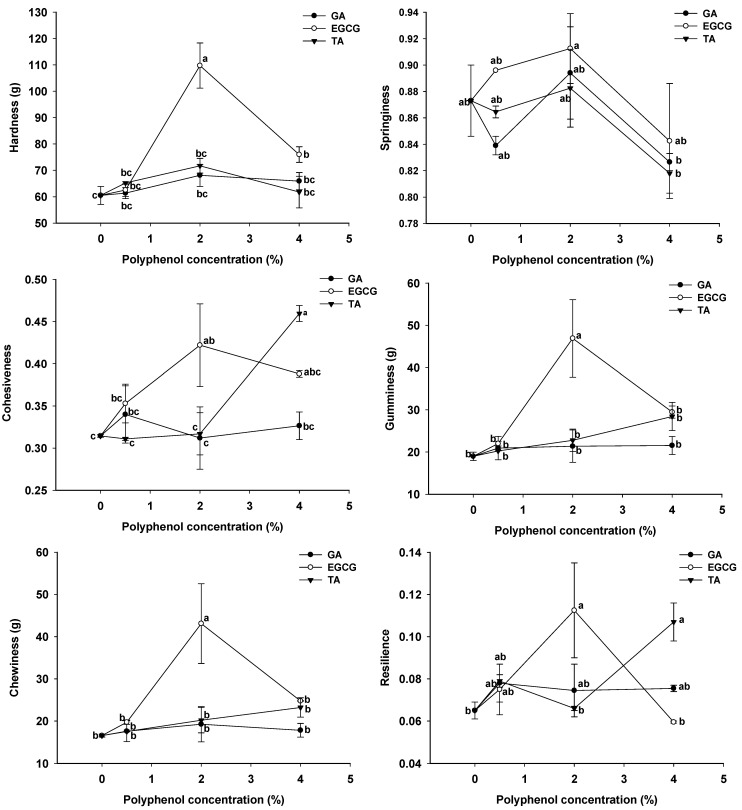
Texture profile analysis of the phenolic acid-modified ginkgo seed protein gels at different concentrations (0, 0.5, 2.0, 4.0%, *w/w*) of gallic acid (GA), epigallocatechin gallate (EGCG), and tannic acid (TA). Values without a common letter were significantly different (*p* < 0.05).

The texture of gel-based foods is a significant quality characteristic that influences the flavor, mouthfeel, and swallowing experiences [46]. As shown in Figure 6, GA and TA had little effect on the texture of the GSPI gels, except that 4.0% of TA significantly increased gel cohesiveness and resilience (*p* < 0.05). EGCG, at 2.0%, greatly promoted the hardness, cohesiveness, gumminess, chewiness, and resilience of the gels (*p* < 0.05). However, such effects diminished when the EGCG concentration was increased to 4.0%. The texture profiles were consistent with the rheological properties of the gels (Figure 4). EGCG is known to have a strong protein binding affinity [35]. EGCE can strengthen the structure of GSPI gel by forming hydrogen bonding and hydrophobic interactions [47]. In addition, the base-induced oxidation of phenolic acids produced quinones, which functioned as bridges to generate protein polymers via thiol-quinone or amine-quinone additions [48].

### 3.7. Microstructure of GSPI-Phenolic Acid Gels

**Figure 7:**
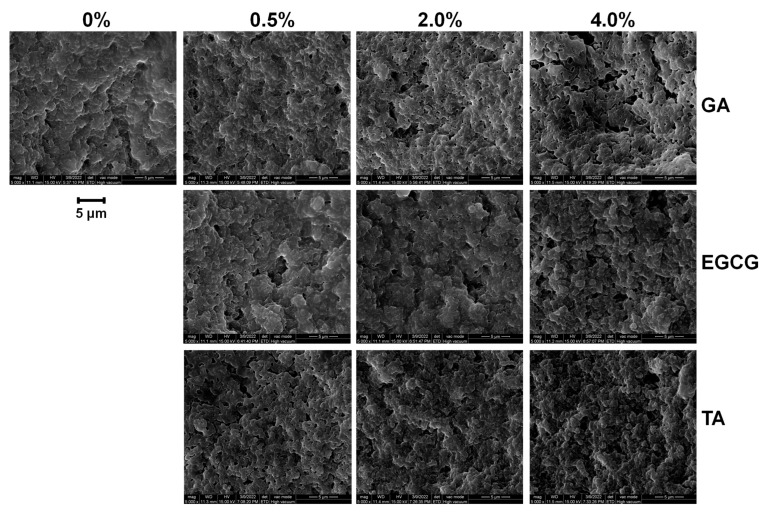
Cross-section of the phenolic acid-modified ginkgo seed protein gels under scanning electron microscopy with an amplification level of 5000× *g*.

SEM was used to study the microstructure of the GSPI gels. As shown in Figure 7, GSPI formed a particle gel network following thermal treatment. The weak electrostatic repulsion between GSPI particles resulted in a granular structure upon heating [33]. This explained the poor WHC of the GSPI gels (Figure 5B). The phenolic acid-treated gels exhibited a denser, more homogeneous matrix than that of the control gel, likely due to polyphenol-induced protein unfolding and cross-linking [48]. This compact structure resisted destructive forces, which explained the enhanced texture characteristics (Figure 6) and rheological properties (Figure 4).

### 3.8. Fourier Transform Infrared (FT-IR) Spectrum

**Figure 8:**
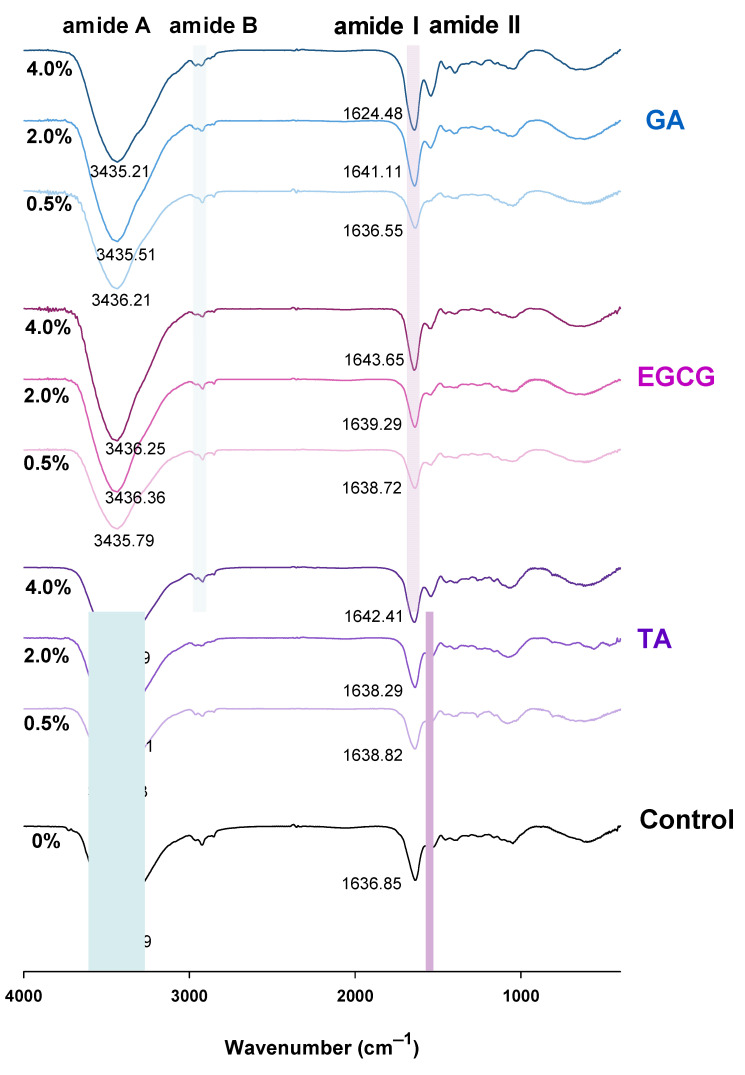
Fourier transform infrared spectrum of the phenolic acid-modified ginkgo seed protein gels at different concentrations (0, 0.5, 2.0, 4.0%, *w/w*) of gallic acid (GA), epigallocatechin gallate (EGCG), and tannic acid (TA).

FT-IR spectroscopy was used to characterize protein structural changes and chemical groups. Figure 8 shows the FT-IR spectra of the GSPI-phenolic acid gels. A major amide A peak was observed at 3200–3600 cm^−1^ for all gels, which corresponded to the intermolecular N-H and O-H stretching vibrations of the GSPI and phenolic acids [49]. This peak shifted towards higher wavenumbers following phenolic acid treatments, indicating protein–polyphenol interactions via hydrogen bonding. The amide B band at 2910–2940 cm^−1^, caused by the asymmetrical stretching of CH_2_ and NH_3_^+^, shifted to lower wavenumbers after the phenolic acid treatments. The *ε*-amino group of lysine, a nucleophilic residue, can readily react with oxidized phenolics. This may explain the shift of the amide B band due to a decrease in free NH_3_^+^ groups from lysine residues [50]. Changes in protein secondary structures were closely related to the amide I at 1640 cm^−1^ and amide II at 1540 cm^−1^ bands. The amide I band was caused by the C=O double bond stretching vibration, while the amide II band was caused by the C-N stretching and N-H bending [51]. The shift of these bands to higher wavenumbers indicated changes in protein secondary structures. Moreover, a shift of the amide I band towards the high wave region indicated a higher bound water content in the gel [49], which aligns with the WHC results (Figure 5B).

## 4. Conclusions

In this study, it was found that alkali-induced oxidized phenolic acids could be used to facilitate the formation of a continuous GSPI gel network by altering the protein tertiary structure and inducing covalent cross-linking of proteins. The effects of phenolic acids were different depending on the type and concentration of the phenolic acids. In the case of the GSPI gels, EGCG, at a moderate concentration (2.0%), was the most effective in promoting the viscoelastic properties, WHC, and texture properties of the gels. As a result of elucidating the interactions between phenolic acids and GSPI, our study provides a convenient method for improving GSPI-based gelled foods and plant protein-derived products, in general, by understanding the interaction between these two compounds.

## Data Availability

Data is contained within the article.
